# Preconditioning in an Inflammatory Milieu Augments the Immunotherapeutic Function of Mesenchymal Stromal Cells

**DOI:** 10.3390/cells8050462

**Published:** 2019-05-15

**Authors:** Luis A. Rodriguez, Arezoo Mohammadipoor, Lucero Alvarado, Robin M. Kamucheka, Amber M. Asher, Leopoldo C. Cancio, Ben Antebi

**Affiliations:** 1 United States Army Institute of Surgical Research, San Antonio, TX 78234, USA; luis.a.rodriguez739.ctr@mail.mil (L.A.R.II); arezoo.mohammadipoor.ctr@mail.mil (A.M.); lucero.alvarado@outlook.com (L.A.); robin.m.kamucheka.ctr@mail.mil (R.M.K.); amber.m.asher@gmail.com (A.M.A.); leopoldo.c.cancio.civ@mail.mil (L.C.C.); 2 Oak Ridge Institute for Science and Education, Oak Ridge, TN 37830, USA; 3 University of Texas at San Antonio, San Antonio, TX 78249, USA

**Keywords:** adipose tissue, bone marrow, mesenchymal stromal cells (MSCs), hypoxia, Cytomix, immunomodulation, anti-inflammatory

## Abstract

Multipotent mesenchymal stromal cells (MSCs) have emerged as potent therapeutic agents for multiple indications. However, recent evidence indicates that MSC function is compromised in the physiological post-injury milieu. In this study, bone marrow (BM)- and adipose-derived (AD)-MSCs were preconditioned in hypoxia with or without inflammatory mediators to potentiate their immunotherapeutic function in preparation for in vivo delivery. Human MSCs were cultured for 48 h in either normoxia (21% O_2_) or hypoxia (2% O_2_) with or without the addition of Cytomix, thus creating 4 groups: (1) normoxia (21%); (2) Cytomix-normoxia (+21%); (3) hypoxia (2%); and (4) Cytomix-hypoxia (+2%). The 4 MSC groups were subjected to comprehensive evaluation of their characteristics and function. Preconditioning did not alter common MSC surface markers; nonetheless, Cytomix treatment triggered an increase in tissue factor (TF) expression. Moreover, the BM-MSCs and AD-MSCs from the +2% group were not able to differentiate to chondrocytes and osteoblasts, respectively. Following Cytomix preconditioning, the metabolism of MSCs was significantly increased while viability was decreased in AD-MSCs, but not in BM-MSCs. MSCs from both tissues showed a significant upregulation of key anti-inflammatory genes, increased secretion of IL-1 receptor antagonist (RA), and enhanced suppression of T-cell proliferation following the Cytomix treatment. Similarly, following a lipopolysaccharide challenge, the Cytomix-treated MSCs suppressed TNF-α secretion, while promoting the production of IL-10 and IL-1RA. These preconditioning approaches facilitate the production of MSCs with robust anti-inflammatory properties. AD-MSCs preconditioned with Cytomix under normoxia appear to be the most promising therapeutic candidates; however, safety concerns, such as thrombogenic disposition of cells due to TF expression, should be carefully considered prior to clinical translation.

## 1. Introduction

Multipotent mesenchymal stromal cells (MSCs) have become a popular tool for treating a myriad of immune- and inflammatory-mediated conditions, such as multiple sclerosis, osteoarthritis, inflammatory bowel disease, and acute respiratory distress syndrome (ARDS), due to their immunomodulatory, anti-inflammatory, antimicrobial, and anti-apoptotic effects [1,2]. MSCs can be isolated from various tissues, but are most frequently isolated from bone marrow (BM) [3]. However, BM aspiration is a painful and invasive procedure. In contrast, adipose-derived (AD) MSCs (AD-MSCs) can be easily and non-invasively harvested from subcutaneous adipose tissue. AD-MSCs have been described to have similar characteristics to BM-MSCs, with respect to plastic adherence, fibroblast-like morphology, and therapeutic properties [4,5,6].

In clinical applications, cell therapies involve a large number of cells (1–10 × 10^6^ cells/kg) in order to elicit a therapeutic effect [7,8,9]. Therefore, in vitro MSC production must be done under optimal culture conditions to maximize cell yield without compromising cell quality. In vitro expansion is typically done under atmospheric, non-physiological oxygen tension (normoxia; 21% O_2_), which has been shown to impair cell function [10]. Interestingly, both BM-MSCs and AD-MSCs reside in low oxygen environments in the body. In the BM, oxygen levels range from 1.5% to 4.2% [11], while in adipose tissue, oxygen levels vary from 4.7% to 8.9% [12]. Indeed, BM-MSCs and AD-MSCs have been shown to have increased ‘stemness’ when cultured under hypoxic conditions in vitro [10,13,14,15,16].

In patients with pulmonary diseases, such as ARDS, systemic oxygen levels are intrinsically hypoxic due to impaired alveolar gas exchange [17]. Additionally, the exacerbated inflammatory systemic response, characteristic of ARDS patients, is manifested, in part, by elevated levels of inflammatory mediators, such as interleukin (IL)-1β, tumor necrosis factor (TNF)-α, and interferon (IFN)-γ [18]. For MSC therapy, these physiological insults potentially jeopardize cell engraftment, viability, and overall therapeutic response [1]. One approach that has been investigated as a potential technique for preparing cells for these physiological insults is in vitro preconditioning. Preconditioning may prepare the cells for the harsh, injurious environment they will experience upon in vivo administration. Additionally, preconditioning approaches have been successfully used to augment the therapeutic function of MSCs [19,20,21]. Notably, both hypoxia and inflammatory cytokines have been explored for preconditioning cells [22,23,24].

While preconditioning has been shown to successfully augment cell characteristics and function, it is not without safety concerns. Preconditioning may negatively alter cell function and potentially cause, among other things, over-expression of tissue factor (TF). TF is a cell surface protein that is important in the initiation of blood coagulation. Studies have shown that under certain conditions MSCs express TF, which may increase the risk of thrombosis following administration [25,26,27,28,29].

**Figure 1:**
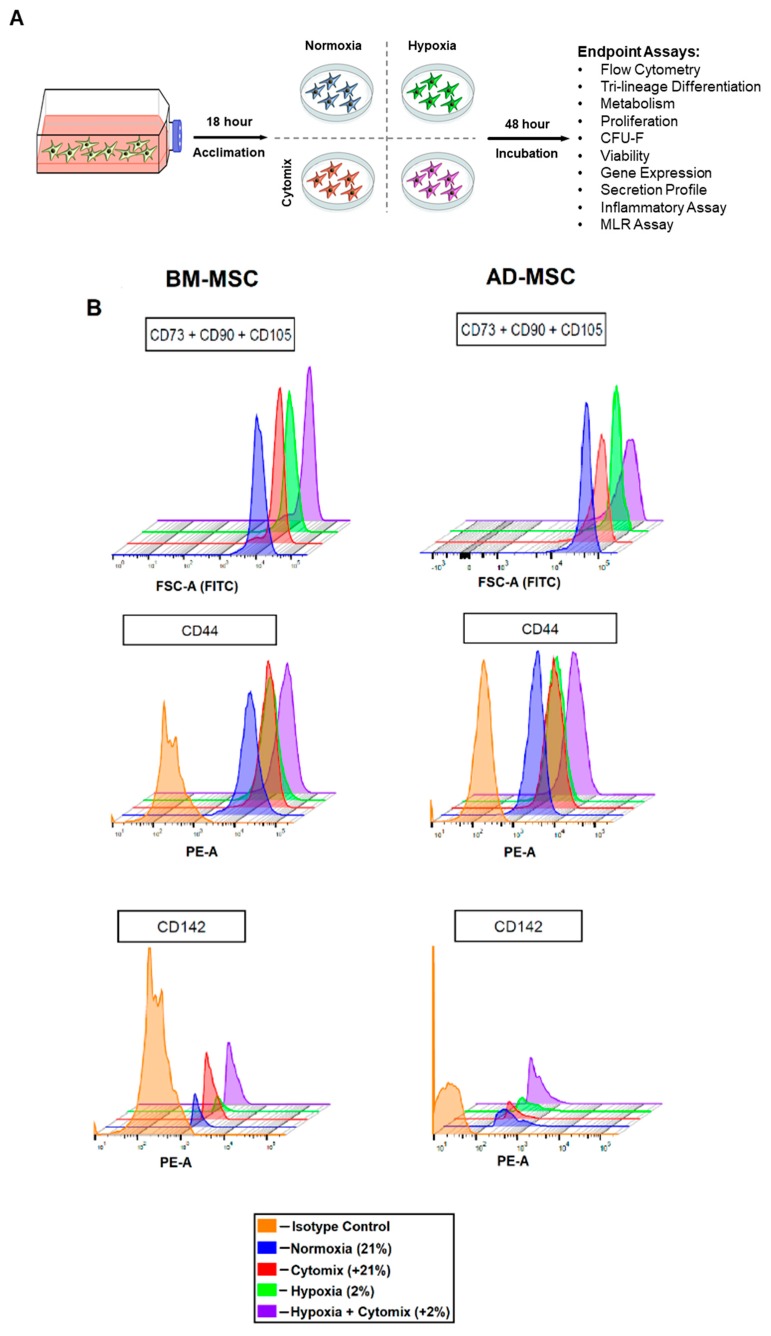

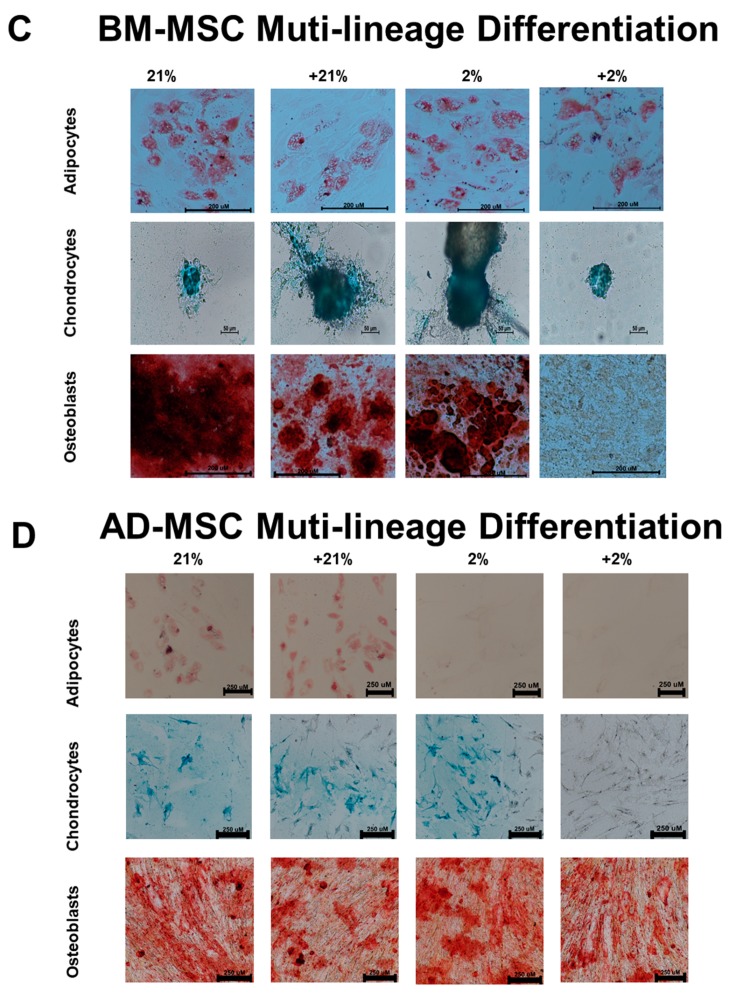
Experimental design, surface marker expression, and tri-lineage differentiation of preconditioned bone marrow (BM) and adipose (AD) derived mesenchymal stromal cells (MSCs). (**A**) MSCs were acclimated for 18 h after thawing and then incubated under normoxia or hypoxia with or without the addition of Cytomix. Following 48-hour incubation, endpoint analyses were conducted. (**B**) Tissue factor (TF) surface expression was increased in BM-MSCs after Cytomix treatment and in Cytomix-hypoxia-treated AD-MSCs. (**C**) BM-MSCs from the Cytomix-hypoxia group were unable to differentiate down the osteogenic pathway. (**D**) AD-MSCs were unable to differentiate into adipocytes under hypoxia or chondrocytes following the Cytomix-hypoxia treatment. Osteogenesis evaluation was done by alizarin red staining; adipogenesis was performed by oil red O staining; and chondrogenesis was conducted by alcian blue staining.

In this study, a combination of hypoxia and inflammatory mediators was used to precondition MSCs. The rationale was that these conditions are physiologically relevant and may therefore augment their therapeutic potency for in vivo administration. To the authors’ knowledge, this is the first study to use the combination of pro-inflammatory cytokines and hypoxia to precondition BM-MSCs and AD-MSCs. Our analysis included a comprehensive evaluation of the characteristics, safety (i.e., *TF* expression), and therapeutic function of MSCs derived from both BM and adipose tissues (Figure 1A). Our aim was to identify the optimal preconditioning approach using the best MSC candidate (BM or AD) for immune- and/or inflammatory-mediated diseases. 

## 2. Materials and Methods

### 2.1. Isolation of BM-MSCs and AD-MSCs

Human BM-MSCs were isolated from commercially available mononuclear cells (MNCs) (AllCells LLC; Emeryville, CA, USA), as previously described [30].

Human AD-MSCs were isolated from consenting patients undergoing abdominoplasty surgery in accordance with protocols reviewed and approved by the U.S. Army Medical Research and Materiel Command Institutional Review Board (H-11-020/M-10128). Briefly, surgically extracted adipose tissue was removed from any connecting tissue and placed in α-MEM media containing 1% antibiotics/antimycotics and 1% fetal bovine serum (FBS) and left in a cell culture hood overnight for next day processing. The adipose tissue was homogenized and washed by centrifugation with Hank’s buffered saline solution (Thermo Fisher Scientific, Waltham, MA). For every 2 mL of displaced fat, 25 mg of Collagenase II (Gibco) was dissolved in HBSS to achieve a concentration of 10 mg/mL. The collagenase/HBSS mixture was purified by filtration (0.22 μm), and 1% FBS and 1% antibiotic/antimycotics were added. The adipose was treated with the collagenase solution for 60 min at 37 °C at 150 rpm using an orbital shaker incubator. After digestion, the digested layer was filtered through 100 µm and 70 µm filters. After filtration, the digested solution was centrifuged at 1900 rpm for 10 min. The resulting cell pellet was re-suspended with α-MEM media and counted. Standard cell culture flasks were seeded at 3 × 10^4^ cells/cm^2^. After overnight culture, the flask was gently tapped, and the media was changed to remove any unwanted cells and/or debris.

### 2.2. Culture Conditions

Passage 2 MSCs (11–15 cumulative population doublings) were cultured for 48 h in either standard oxygen tension (i.e., normoxia; 5% CO_2_/95% air; 37 °C) or hypoxia (2% O_2_/5% CO_2_/93% N_2_; 37 °C) using a dedicated hypoxia station (HypOxystation H35, HypOxygen, Frederick, MD, USA). MSCs in normoxia or hypoxia were cultured with or without the addition of Cytomix, a mixture containing TNF-α, INF-γ, and IL-1β, all at 5 ng/mL (Thermo Fisher Scientific, Waltham, MA, USA), to create 4 experimental groups: 1) normoxia (21%); 2) Cytomix-normoxia (+21%); 3) hypoxia (2%); and 4) Cytomix-hypoxia (+2%).

Unless otherwise noted, all biological assays were run in triplicates from one donor per cell type (either BM-MSCs or AD-MSCs). In the inflammatory assay, three donors per cell type (for a total of 6 donors) were run in triplicates to examine donor-dependent differences and validate results.

### 2.3. BM-MSC and AD-MSC Surface Markers

MSCs were examined by flow cytometry for the expression of common MSC markers. Human MSC Analysis Kit (BD, Biosciences, San Jose, CA, USA) was used for assessing BM-MSCs and AD-MSCs. The MSCs were pooled by cell type and treatment condition (i.e., one sample per cell type per treatment group). Cells were then stained with pre-conjugated antibodies according to manufacturer’s instruction. Briefly, cells were incubated with staining buffer containing 1% bovine serum albumin (BSA) and Fc blocker (BioLegend, San Diego, CA, USA) for 10 min at a cell concentration of 1 × 10^6^/mL in order to reduce non-specific binding. Then, the antibody cocktail for MSC-positive markers (CD90-FITC, CD105-PerCP-Cy5.5, and CD73-APC), and negative markers (CD45-PE, CD34-PE, CD11b-PE, CD19-PE, and HLA-DR-PE) were added to the cells. In separate tubes, the antibody cocktail for MSC-positive markers and PE-conjugated mouse monoclonal CD142-PE antibody (TF and BD, Biosciences, San Jose, CA, USA) and CD44-PE were added to the cells. After 20 min of incubation in the dark, at 22 °C, cells were washed to remove excess antibodies. Acquisition was carried out on a BD FACSCanto II or on a BD FACSCelesta using the BDFACS Diva version 8.0.1.1 software. Analysis was completed using FlowJo version 10 analysis software.

### 2.4. Multi-Lineage Differentiation

Multi-differentiation assay was used to evaluate the capacity of MSCs to give rise to osteoblasts, adipocytes, and chondrocytes using a commercially available differentiation medium (StemPro Differentiation Kits, Thermo Fisher Scientific), as previously described [31]. For this purpose, the MSCs from the different groups were cultured in 8-well chamber slides for histological evaluation.

### 2.5. Colony Forming Unit-Fibroblast (CFU-F) Assay

The colony-forming unit fibroblast (CFU-F) assay was used as an indicator of progenitor cells. Preconditioned MSCs were plated at 100 and 200 cells per well in a six-well plate. Media were changed 3–4 days and the cells were allowed to grow for 7–10 days. Cells were then washed with PBS and fixed with chilled methanol for 10 min at room temperature. The plates were allowed to air-dry and then stained with Giemsa to visualize the colonies. Colonies larger than 50 cells were counted and reported as CFUs/well. Cells were measured in order to provide a semi-quantitative record of morphology changes.

### 2.6. Cell Apoptosis/Viability and Morphology

Cells apoptosis was measured by flow cytometry using Annexin V kit (BioRad, Hercules, CA, USA) per the manufacturer’s instructions. MSCs were collected, washed in PBS containing 1% BSA, and subsequently re-suspended in annexin binding buffer at a cell concentration of 1.5 × 10^6^/mL. MSCs were then incubated for 10 min with annexin V-FITC in dark. Propidium iodide (PI) was added to BM-MSCs to be immediately analyzed by FACSCelesta (BD Biosciences, San Jose, CA, USA) using the BDFACS Diva and FLowJo software. Cell fragments were removed by morphological gating. Cells negative for annexin V-FITC and PI were considered viable; annexin V-FITC-positive and PI-negative cells were considered apoptotic; and annexin V-FITC-positive and PI-positive cells were considered late apoptotic/necrotic.

For a semi-quantitative assessment of cell morphology (i.e., cell length), the MSCs were stained with a fluorescent Live/Dead Cell Viability Kit (Life Technologies, Grand Island, NY, USA) and a Hoechst 33342 nucleic acid stain (Life Technologies), as previously described [23,32]. Cells from each treatment group were measured for their length across five distinct areas using the length measure function in NIS-Elements AR (Nikon, Tokyo, Japan). Measurements were averaged and compared between the 4 groups.

### 2.7. Metabolic and Proliferation Assays

MSCs were evaluated for their metabolic activity using the Vybrant assay (Thermo Fisher Scientific, Waltham, MA, USA), as previously described [30]. DNA concentration was measured using the Quant-iT PicoGreen assay (Invitrogen, Carlsbad, CA, USA) to evaluate cell proliferation, as previously described [23]. Metabolic data were normalized to DNA concentration and are presented as a relative quantity (RQ).

### 2.8. Gene Expression

To determine gene expression via quantitative real-time polymerase chain reaction (qRT-PCR), total RNA was extracted from MSCs using Trizol (Thermo Fisher Scientific) and reverse-transcribed using a High-Capacity cDNA Archive Kit (Applied Biosystems, Foster City, CA, USA), as previously described [30]. The following transcripts were investigated: vascular endothelial growth factor A (*VEGF-A*), factor 3 (*F3*, encodes for TF), prostaglandin-endoperoxide synthase 2 (*PTGS-2*), high-mobility group box 1 (*HMGB-1*), B-cell lymphoma 2 (*BCL-2*), cytochrome c (*CYCS*), *BCL-2* associated protein (*BAX*), caspase 3 (*CASP3*), catalase (*CAT*), heme-oxygenase (*HMOX1*), toll-like receptor 4 (*TLR-4*), tumor necrosis factor-inducible gene 6 (*TNFAIP6*), and stannioclacin-1 (*STC-1*). All assays were done in duplicates and gene expression is expressed as an RQ calculated from ΔΔCt of the sample of interest, where C_T_ is the threshold cycle.

$$\Delta C_{T\;Gene\;of\;Interest}=\;C_{T\;Housekeeping\;}\;-\;C_{T\;Gene\;of\;Interest}$$

$$\Delta \Delta C_{T\;Gene\;of\;Interest}=\;\;\Delta C_{T\;\;Gene\;of\;Interest}-\Delta C_{\begin{array}{c} T\;Reference \end{array}\;}$$

$$RQ=\;2^{-\Delta \Delta C_{T\;Gene\;of\;Interest}}$$

### 2.9. Secretion Profile

The secretory profiles of the BM-MSCs and AD-MSCs were assessed following preconditioning using the cytokine-chemokine multiplex kit (detection range of 0–10,000 pg/mL, Millipore, Billerica, MA) to evaluate INF-γ, monocyte chemoattractant protein (MCP)-1, interleukin-1 alpha (IL-1α), IL-1β, IL-1 receptor antagonist (RA), IL-4, IL-6, IL-8, IL-10, IL-12, and *VEGF-A*. To accomplish this, samples’ supernatants were spun down to remove any remaining cells and placed in −80 °C until simultaneous analysis. Samples were concentrated using an Amicon ultra-2 centrifugal filter unit (MilliporeSigma, Burlington, MA, USA) for 30 min at 4000× *g*. Samples were then run on a BioPlex 200 system (Bio-Rad, Hercules, CA, USA), following the manufacturer’s instructions, as previously described [31]. Data were standardized to the total amount of protein using the Pierce^TM^ 660 nm protein assay (Thermo Scientific, Waltham, MA, USA) and reported as picogram per gram of total protein (pg/g).

### 2.10. Inflammatory Assay

To examine whether the preconditioning approaches were donor-dependent, BM-MSCs and AD-MSCs were investigated from 6 unique donors (three donors per cell type). In this assay, the cells were seeded at 5 × 10^4^ cells per well, in quadruplicates, in a 24-well plate and placed in standard culture conditions for 2 h to allow for cell attachment. MNCs were prepared at a concentration of 0.5 × 10^6^ cells/mL in MNC media. Prior to addition of lipopolysaccharide (LPS), MNC control cells were set aside. Next, LPS was added to the MNCs at a concentration of 50 ng/mL and 0.5 × 10^6^ of the stimulated MNCs were added to the MSCs. Following 18 h incubation, the co-cultures supernatant was collected, cell debris was removed via centrifugation, and the resulting conditioned media (CM) were analyzed for levels of IFN-γ, TNF-α, IL-1RA, IL-1β, and IL-10 using a Milliplex kit.

### 2.11. Mixed Lymphocyte Reaction

BM-MSCs and AD-MSCs were plated at 5 × 10^4^ cells per well, in triplicates, in a 24-well plate. MSCs were incubated for at least 2 h to allow for cell adhesion before adding MNCs to the well. Prior to staining, 0.5 × 10^6^ MNCs were set aside as an unstained control. In the dark, MNCs were brought to a concentration of 10 × 10^6^ cells/ml in PBS. Carboxyfluorescein succinimidyl ester (CFSE; Sigma Aldrich, St. Louis, MO, USA) was used to fluorescently label the MNCs at a concentration of 5 µM per milliliter. The MNCs were mixed well and incubated for five min in the dark. After incubation, the MNCs were washed twice and re-suspended in MNC media (RPMI 1640 medium supplemented with 10% FBS, 1% antibiotics-antimycotics, and 1% L-glutamine) at a concentration of 0.5 × 10^6^ cells/mL. Then, 1 mL of cell suspension was designated as an unstimulated control. The remaining MNCs were stimulated with Phytohaemagglutinin (PHA) at a concentration of 5 µg/mL. Next, 1 mL of stained and stimulated MNCs were added to MSCs and incubated for 96 h. In addition, MNCs were plated in the absence of MSCs as a positive control. After 96 h, the floating MNCs and conditioned media (CM) were collected for further analysis. The collected MNCs were centrifuged and resuspended in filtered PBS containing 1% BSA and FC Block (BioLegend, San Diego, CA, USA). MNCs were incubated for 10 min in dark and subsequently labeled with pre-conjugated CD3-APC antibody (BD Pharminogen, San Jose, CA, USA) for another 15 min. After washing, 7AAD viability dye (BD Pharminogen, San Jose, CA, USA) was added and the cells incubated for 5 min. Proliferation rate of the live CD3-positive T-cells was acquired by FACSCelesta or FACSCanto II (BD Biosciences, San Jose, CA, USA) using the BDFACS Diva and FlowJo software. The CM were analyzed with a Milliplex kit for the following cytokine/chemokines: IL-1α, IL-1β, IL-1RA, IL-4, IL-6, IL-8, IL-10, IL-12, MCP-1, and IFN-γ.

### 2.12. Statistical Analysis

Results are presented as means ± standard errors of the mean. Unless otherwise stated, all assays were run in triplicates from one donor per cell type. All statistical tests were performed with the aid of GraphPad Prism version 7.01. Experimental data were analyzed with a two-way analysis of variance (ANOVA) followed by Tukey’s multiple comparison post-test. Evaluation and exclusion of outliers was performed using the ROUT method; a probability (*p*) value less than 0.05 was considered statistically significant.

## 3. Results

### 3.1. Surface Marker Expression and Differentiation

To assess whether any of the preconditioning approaches altered cell phenotype, the MSCs were subjected to flow cytometry and multi-differentiation assays. In BM-MSCs, positive markers remained above 97% for all treatment groups. TF (CD142) expression was minimal under normoxia (2.0%) and hypoxia (1.2%). However, following exposure to Cytomix, TF expression dramatically increased under both normoxia (7.8%) and hypoxia (6.6%) (Figure 1B). In AD-MSCs, positive marker expression remained above 93% in all groups. Basal expression of TF was high under normoxia (9.28% for normoxia) and further increased in the Cytomix-hypoxia group (14.8%) (Figure 1B).

Differentiation potential of BM-MSCs into adipocytes and chondrocytes was not affected by any of the treatments; however, the combination of hypoxia and Cytomix inhibited their ability to differentiate down the osteogenic lineage (Figure 1C). AD-MSCs were able to differentiate into osteocytes under all conditions, although mineralization capacity was not as robust as their BM counterparts. Adipocytes were only present in cells exposed to normoxia, whereas hypoxia inhibited their differentiation potential. Chondrogenesis occurred under all conditions except for the Cytomix-hypoxia combination group (Figure 1D).

### 3.2. Cell Metabolism, Proliferation, Clonogenicity, Viability, and Morphology

**Figure 2:**
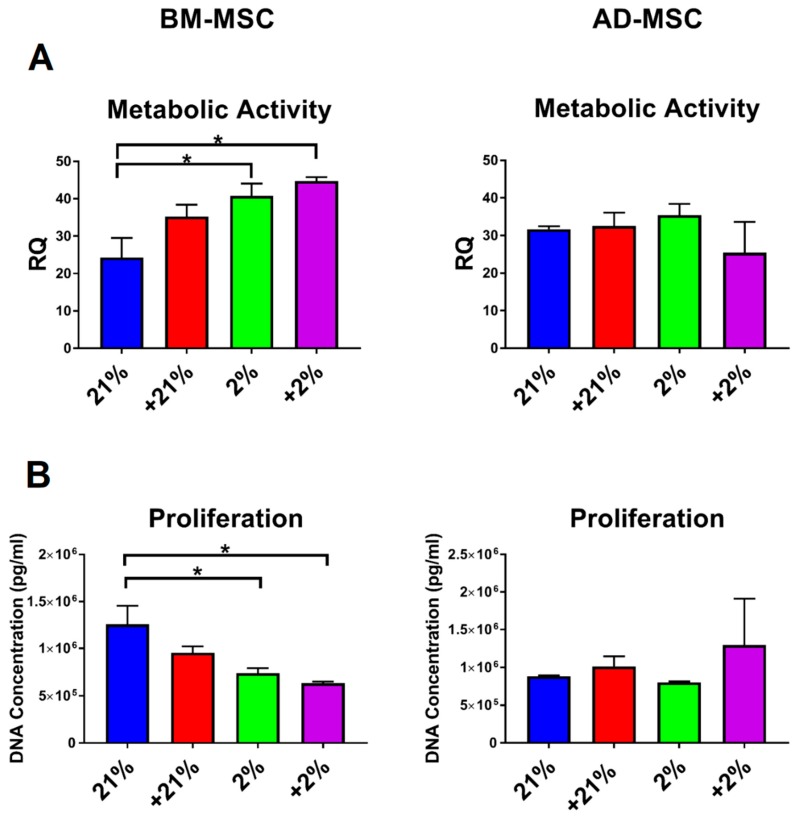

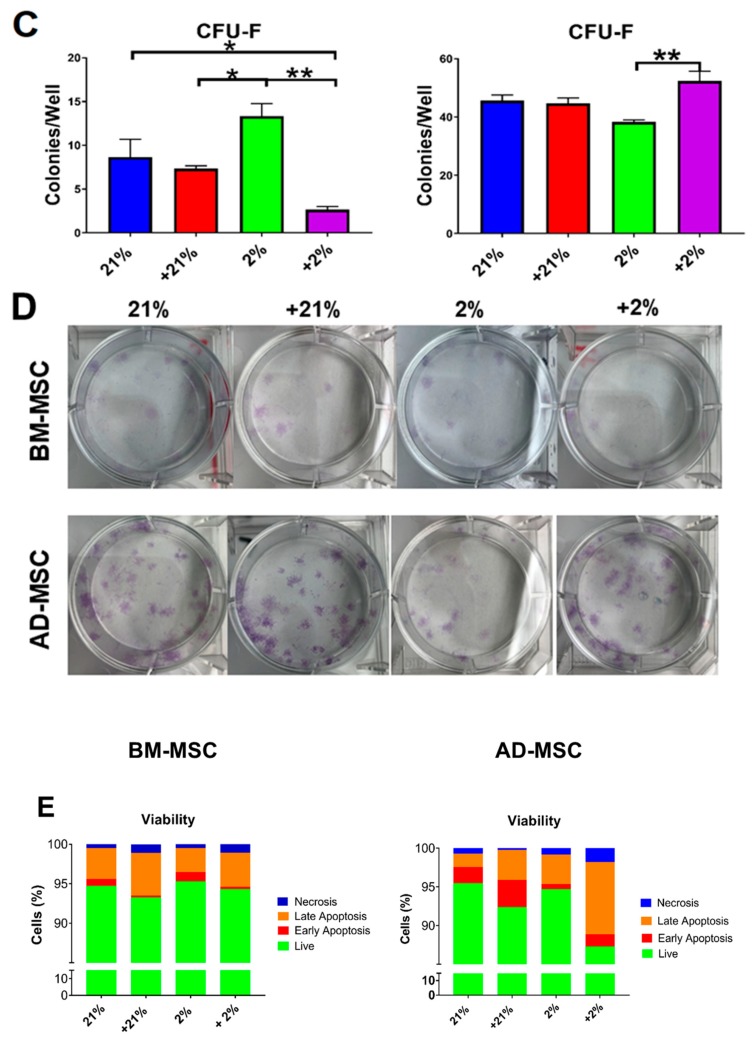

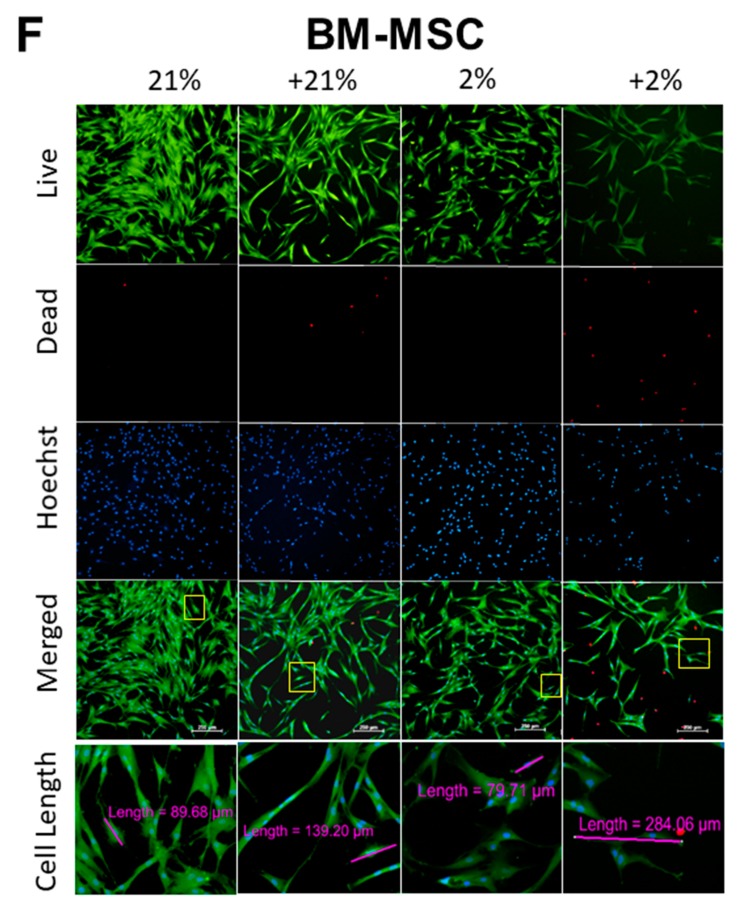

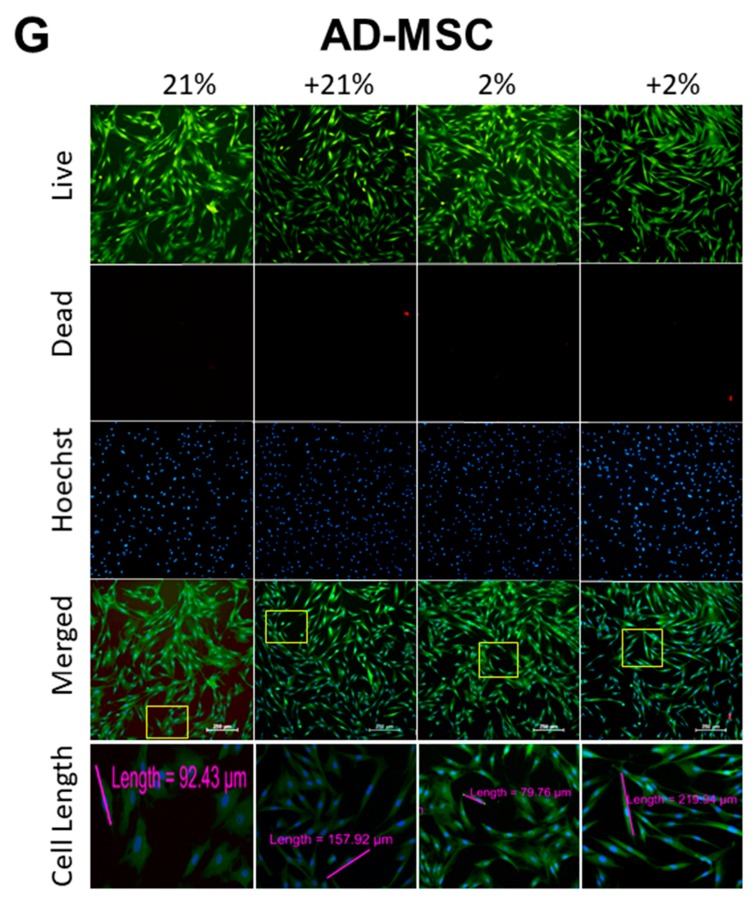

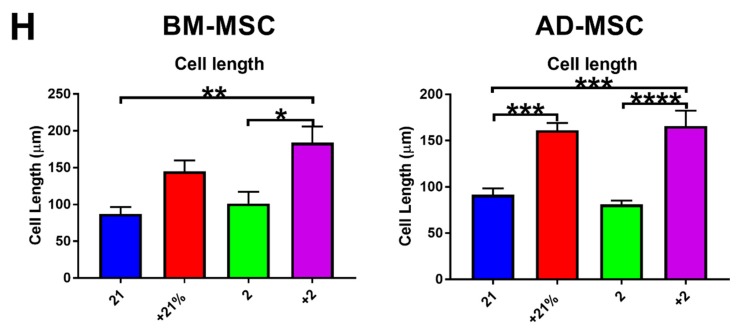
Functional characteristics of preconditioned MSCs. (**A**) Metabolic activity of BM-MSCs significantly increased under hypoxia, while AD-MSCs had no change. (**B**) Proliferative capacity of BM-MSCs was decreased under hypoxia, while AD-MSCs remained unchanged. (**C**) BM-MSCs exhibited increased clonogenic capacity under Cytomix-hypoxia treatment (*p* < 0.05), while the clonogenic capacity of AD-MSCs were unchanged. (**D**) Representative images of colony forming unit assay for each experimental grouped stained with Giemsa. (**E**) Viability was decreased in preconditioned AD-MSCs compared to that in their BM counterparts. (**F**,**G**) Cytomix treatment induced morphological changes in both BM-MSCs and AD-MSCs, including representative images of length measurements. MSCs treated with Cytomix-normoxia and Cytomix-hypoxia appeared more elongated compared to normoxia and hypoxia groups. (**H**) Cell length measurements from fluorescence imaging. The addition of Cytomix made MSCs become more elongated and spindle-like. * *p* < 0.05, ** *p* < 0.01, *** *p* < 0.001, **** *p* < 0.0001. All scale bars represent 250 μM. Viable cells were stained with calcein AM; dead cells were stained with ethidium homodimer-1; and nuclei of all cells were stained with Hoechst.

Viability and functional cell characteristics were also assessed under the different preconditioning approaches. The metabolic activity of BM-MSCs was significantly (*p* < 0.05) increased under hypoxic conditions (hypoxia and Cytomix-hypoxia), whereas the metabolic activity of AD-MSCs was not affected by any of the preconditioning approaches (Figure 2A). Similarly, the proliferative capacity for BM-MSCS was significantly (*p* < 0.05) diminished under hypoxic conditions (hypoxia and Cytomix-hypoxia), whereas AD-MSC proliferation was unaffected by any of the treatment conditions (Figure 2B). Compared to hypoxia preconditioning, the clonogenic capacity of BM-MSCs was significantly decreased (*p* < 0.01) following Cytomix-hypoxia treatment, while in AD-MSCs it was significantly increased (*p* < 0.01; Figure 2C,D).

In BM-MSCs, viability was similar under normoxic (94.75%) and hypoxic (95.3%) conditions. Cytomix treatment slightly decreased MSC viability with concomitant increase in cell apoptosis and necrosis. Similar trends were seen in AD-MSCs, although viability was more significantly affected. Following Cytomix treatment, viability dropped from 95.5% (normoxia) and 94.7% (hypoxia) to 92.4% Cytomix-normoxia) and 87.3% (Cytomix-hypoxia), respectively (Figure 2E). Fluorescent Live/Dead and Hoechst staining demonstrated similar morphological changes in BM-MSCs and AD-MSCs. Most notably, the Cytomix treatment caused cells to become increasingly elongated, compared to the normoxia- and hypoxia-treated cells (Figure 2F,G). Following the addition of Cytomix, BM-MSCs increased in length from 87.3 ± 9.2 μm to 145.1 ± 14.8 μm (*p* = 0.08) under normoxia and from 101.4 ± 15.9 μm to 184.4 ± 21.5 μm (*p* < 0.05) under hypoxia. Similarly, following Cytomix treatment, the length of AD-MSCs increased from 91.8 ± 6.7 μm to 161.5 ± 7.5 μm (*p* << 0.001) under normoxic conditions and from 81.0 ± 4.3 μm to 165.9 ± 16.5 μm (*p* < 0.0001) under hypoxia (Figure 2H).

### 3.3. Gene Expression

**Figure 3:**
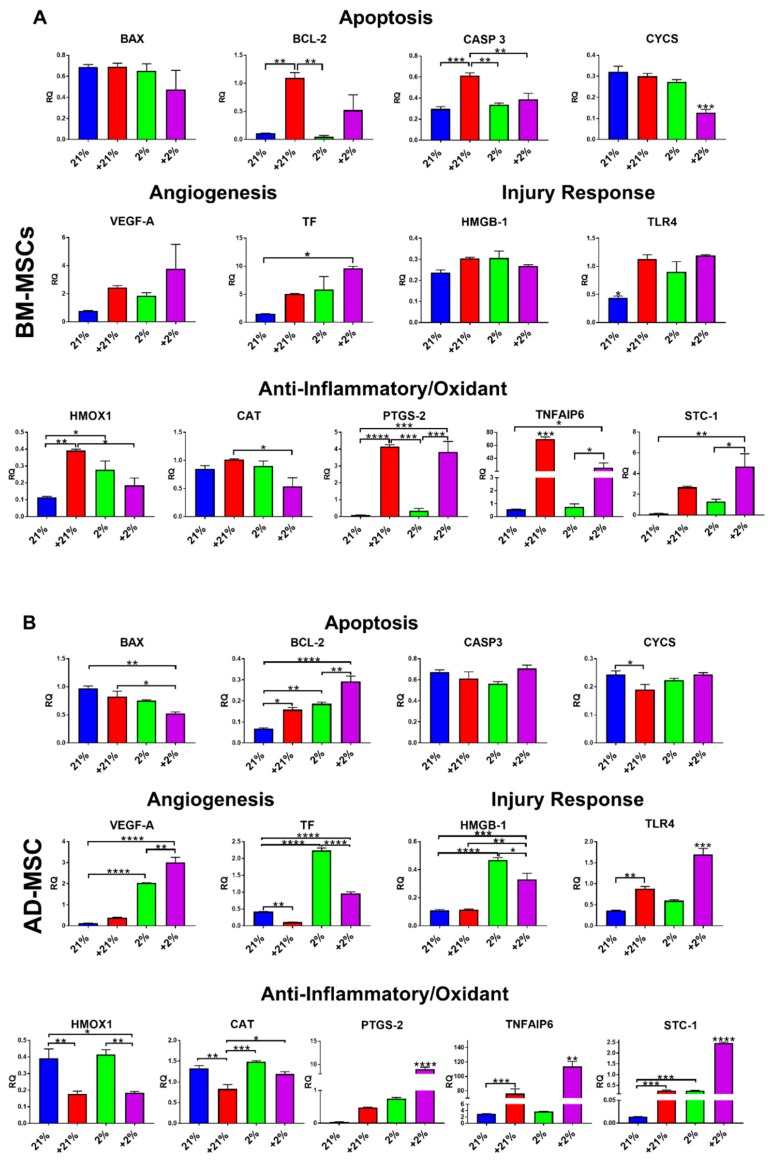
Gene expression of preconditioned MSCs. (**A**) In BM-MSCs, Cytomix-normoxia treatment significantly upregulated gene expression of *BCL-2*, *CASP-3, CYCS*, *TLR-4*, *HMOX1*, *PTGS-2*, and *TNFAIP6*. Hypoxia preconditioning increased *TLR-4* and *HMOX1* gene expression, while Cytomix-hypoxia combination significantly upregulated *TLR-4*, *PTGS-2*, *TNFAIP6*, and *STC-1* and downregulated *CYCS*. (**B**) In AD-MSCs, Cytomix-normoxia treatment significantly upregulated gene expression of *BCL-2*, *TLR-4*, and *TNFAIP6*. Hypoxia preconditioning increased *BCL-2*, *VEGF-A*, *HMGB-1*, *CAT*, *STC-1*, and *HMOX1* gene expression, while Cytomix-hypoxia treatment significantly upregulated *BCL-2*, *VEGF-A*, *TF*, *HMGB-1*, *TLR-4*, *PTGS-2*, *TNFAIP6*, and *STC-1* and downregulated *HMOX1*. * *p* < 0.05, ** *p* < 0.01, *** *p* < 0.001, **** *p* < 0.0001.

Genes related to apoptosis, angiogenesis, injury response, and anti-inflammatory/oxidant properties were evaluated in MSCs following preconditioning and compared to those related to normoxia (21%). In BM-MSCs, the apoptosis-related genes *BCL-2* and *CASP3* were significantly (*p* < 0.01 and *p* < 0.001, respectively) upregulated in response to Cytomix treatment alone, while *CYCS* was downregulated (*p* < 0.001) in the Cytomix-hypoxia group. *TF* gene expression was significantly upregulated (*p* < 0.05) in the Cytomix-hypoxia cells. Expression of *TLR4* was significantly upregulated in all preconditioning groups (*p* < 0.05). *HMOX1* expression was upregulated with Cytomix (*p* < 0.01) and hypoxia (*p* < 0.05). *PTGS-2*, *TNFAIP6*, and *STC-1* expression was significantly upregulated in response to Cytomix (Figure 3A).

AD-MSCs were also affected by the various preconditioning methods, when compared to normoxia. *BAX* expression was downregulated in the +2% group (*p* < 0.01), whereas *BCL-2* was significantly upregulated following all preconditioning approaches, but most prominently in the Cytomix-hypoxia group (*p* < 0.0001). *CYCS* was significantly downregulated (*p* < 0.05) in the Cytomix group only. Similar to BM-MSCs, AD-MSCs exhibited significant upregulation in the angiogenic-related genes *VEGF-A* and *F3* under hypoxia. Unlike in BM-MSCs, *HMGB-1* was significantly upregulated in AD-MSCs in response to hypoxia while *TLR4* was upregulated in response to Cytomix treatment. *HMOX1* and *CAT* were significantly downregulated following Cytomix treatment. *PTGS*, *TNFAIP6*, and *STC-1* were significantly upregulated under all preconditioning methods, but most were dramatically upregulated in the Cytomix-hypoxia group (*p* < 0.0001). (Figure 3B).

### 3.4. Secretion Profile

**Figure 4:**
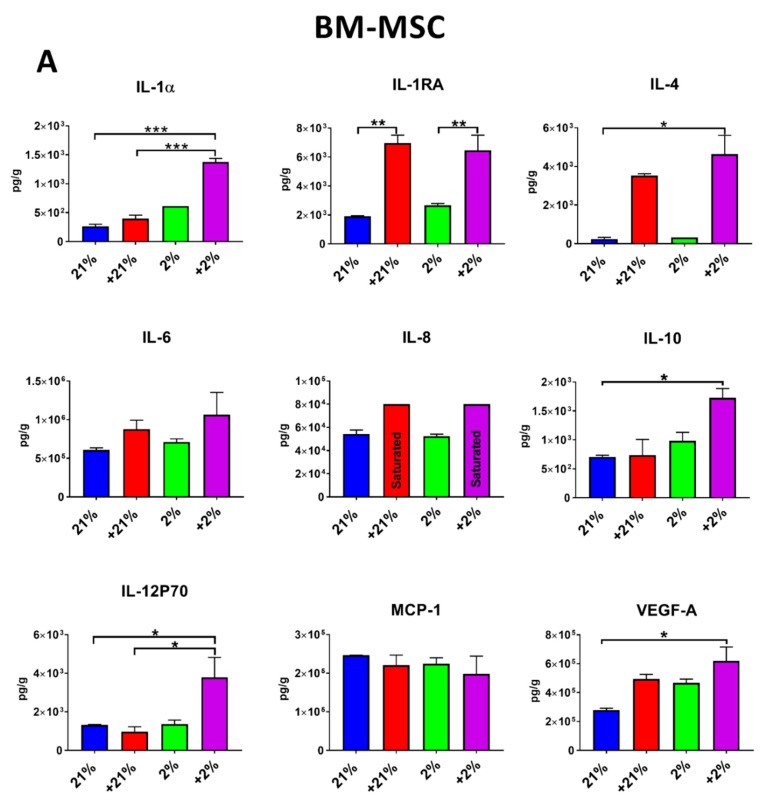

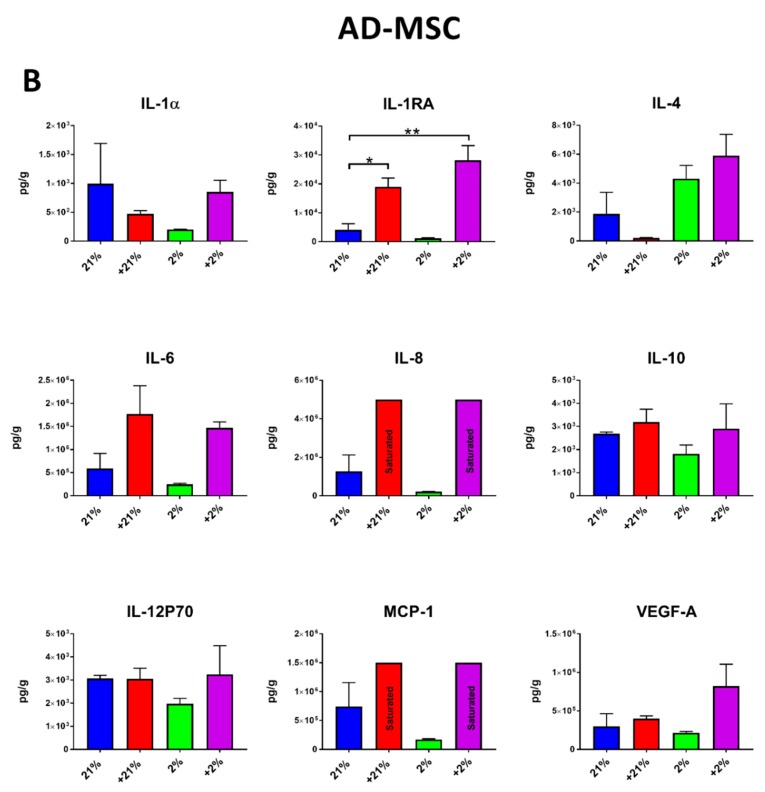
Secretion profile of preconditioned MSCs. (**A**) In BM-MSCs, IL-1RA and IL-8 were increased in Cytomix-treated cells. (**B**) In AD-MSCs, similar to in BM-MSCs, increases were seen in levels of IL-1RA and IL-8 following Cytomix treatment, as well as an increase in MCP-1 secretion. In addition, Cytomix-hypoxia preconditioning yielded further increases in levels of IL-1α, IL-4, IL-10, and IL-12. * *p* < 0.05, ** *p* < 0.01, *** *p* < 0.001.

Since paracrine activity is a key mechanism of action of MSCs, secretion of bioactive factors was evaluated in response to preconditioning and compared to normoxia. In BM-MSCs, secretion of the anti-inflammatory cytokines IL-1RA (*p* < 0.01), IL-4 (*p* < 0.05), and IL-10 (*p* < 0.05), as well as the pro-inflammatory cytokines IL-1α (*p* < 0.001), IL-12 (*p* < 0.05), and IL-8 (saturated level), was significantly increased in the Cytomix-hypoxia group (Figure 4A). IL-1RA secretion also significantly increased in the Cytomix-normoxia group (*p* < 0.01). 

Similar to that by BM-MSCs, secretion of IL-1RA, IL-6, and IL-8 by AD-MSCs was increased following Cytomix treatment, whereas secretion of IL-4 was increased (although not statistically significant) following hypoxia and Cytomix-hypoxia preconditioning (Figure 4B).

### 3.5. Inflammatory Assay

To validate that our findings were due to the preconditioning approaches and not donor-dependent, the anti-inflammatory function of MSCs from different donors (3 BM donors, 3 AD donors, *n* = 6 in total) was evaluated in a co-culture system of MSCs and MNCs stimulated with LPS.

**Figure 5:**
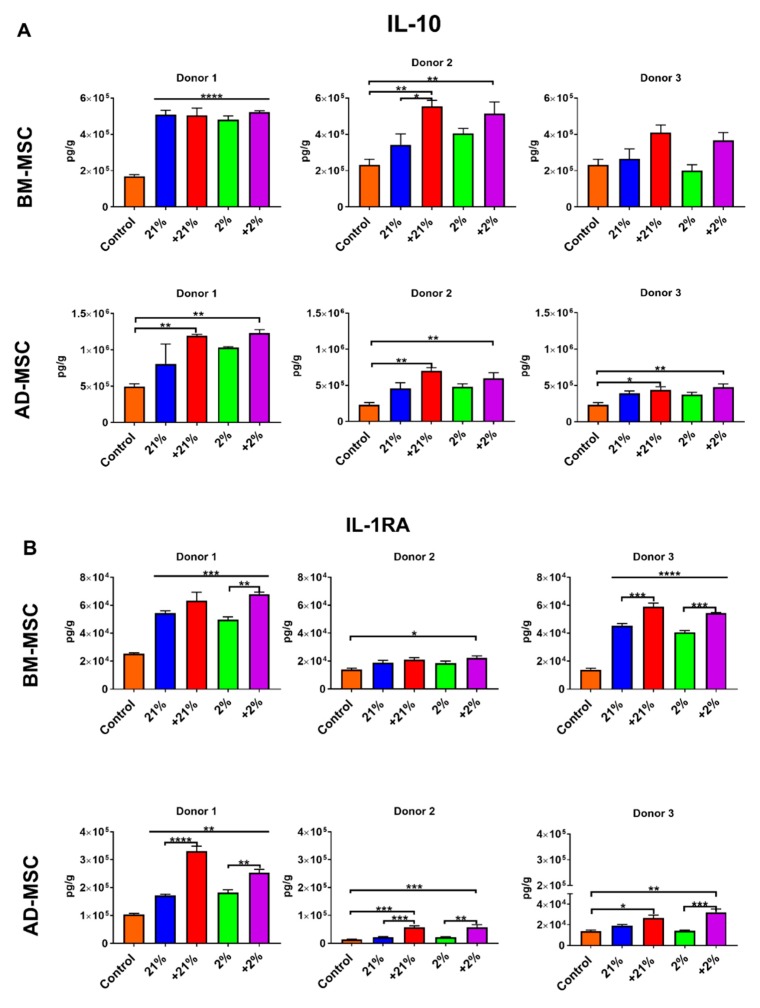

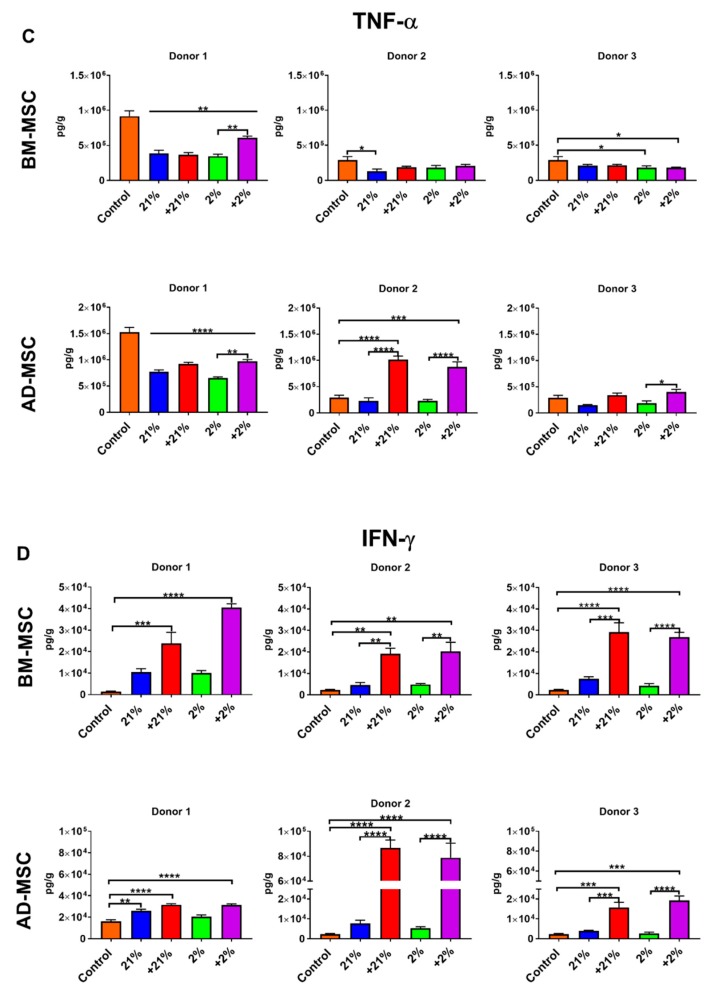

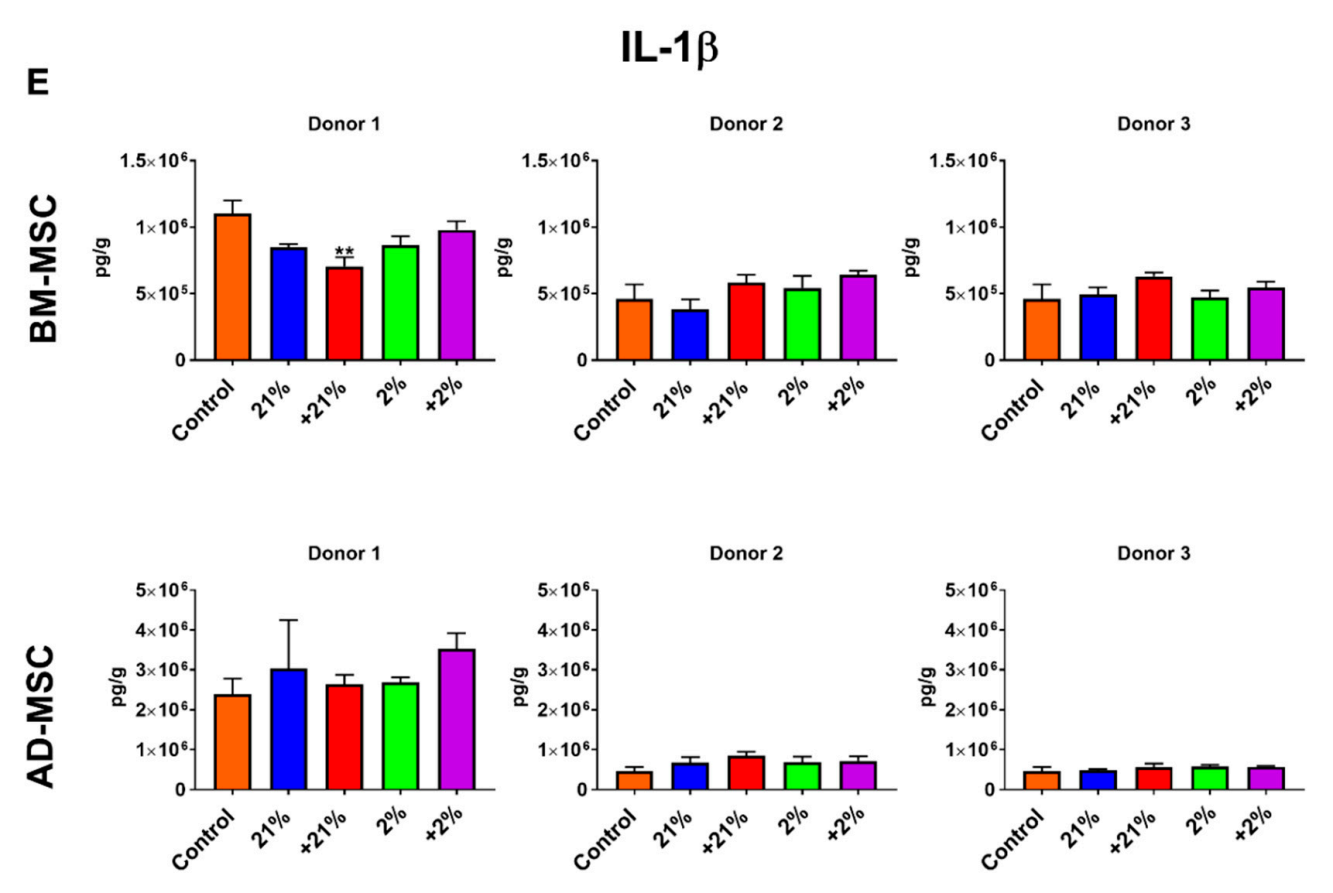
An inflammatory response to a lipopolysaccharide (LPS) challenge in a co-culture system of MSCs and MNCs. Preconditioning significantly augmented the anti-inflammatory response of BM-MSCs and AD-MSCs by increasing IL-10 (**A**) and IL-1RA (**B**) levels while significantly suppressing the secretion of TNF-α (**C**). Cytomix treatment elicited the most potent response with significantly increased production of IFN-γ (**D**), whereas no significant differences were observed in levels of IL-1β (**E**). * *p* < 0.05, ** *p* < 0.01, *** *p* < 0.001, **** *p* < 0.0001.

In BM-MSCs, the secretion of the anti-inflammatory cytokine IL-10 was significantly increased in the Cytomix-normoxia (*p* < 0.01) and Cytomix-hypoxia (*p* < 0.01)-treated groups in donor 2. Donor 1 had high basal secretion of IL-10 and preconditioning did not further increase the secretion capacity. Although failing to meet significance, donor 3 showed a similar response to preconditioning compared to donor 2 (Figure 5A). IL-1RA secretion was significantly affected by preconditioning. Donor 1 showed increased secretion in all groups (*p* < 0.001) while revealing Cytomix-hypoxia futher increased IL-1RA compared to hypoxia (*p* < 0.01). Donor 2 showed an increased in secretion in the Cytomix-hypoxia group (*p* < 0.05). Donor 3 showed IL-1RA secretion increased in all the groups (*p* < 0.0001); however, Cytomix-treated groups had higher secretion than their counterparts (*p* < 0.001) (Figure 5B). TNF-α was significantly suppressed in all groups in donor 1. Donor 2 saw a decrease in the normoxia group while donor 3 showed suppression in hypoxia and Cytomix-hypoxia groups (Figure 5C). INF-γ production followed a similar trend in all donors. Secretion was increased in Cytomix-normoxia (*p* < 0.01) and Cytomix-hypoxia (*p* < 0.01) (Figure 5D). Similarly, IL-1B secretion was largely unaffected by preconditioning (Figure 5E).

In AD-MSCs, IL-10 was increased by preconditioning. In all three donors, Cytomix-normoxia and Cytomix-hypoxia treatment caused a significant increase in secretion (*p* < 0.05 and *p* < 0.01, respectively, Figure 5A). IL-1RA was also significantly affected by preconditioning. All donors showed a similar response, significantly increasing secretion after Cytomix-normoxia and Cytomix-hypoxia (Figure 5B). TNF-α secretion varied between donors. Donor 1 showed all groups suppressed TNF-α secretion. Donors 2 and 3 showed increases in TNF-α compared to controls but the level of secretion was comparable to that in donor 1 (Figure 5C). Similar to their BM counterparts, AD-MSCs from all donors showed significant increases in IFN-γ secretion after Cytomix-normoxia and Cytomix-hypoxia treatment (*p* < 0.001 and *p* < 0.001, respectively; Figure 5D). IL-1B was not affected by preconditioning. 

### 3.6. Mixed Lymphocyte Reaction

**Figure 6:**
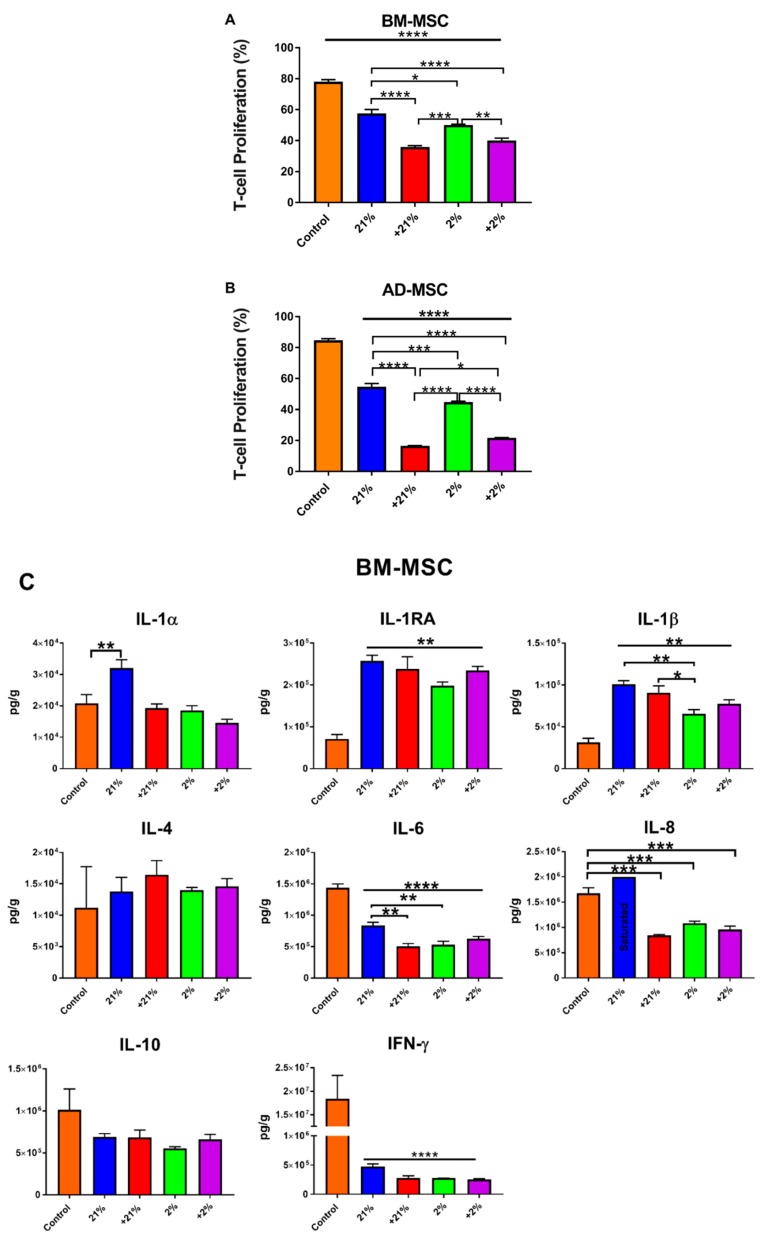

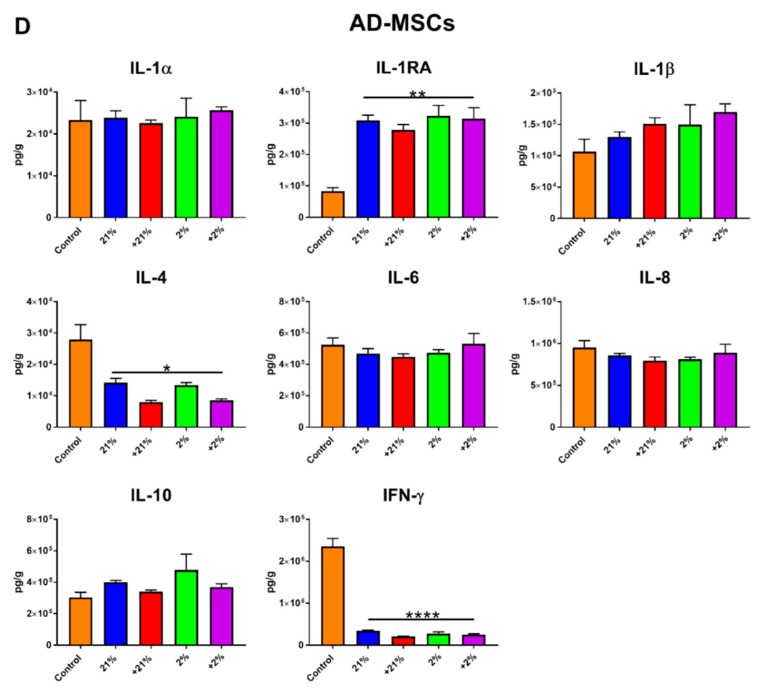
Immunomodulatory function of preconditioned MSCs demonstrated via an MLR assay. (**A**) Preconditioning significantly augmented the immunosuppressive function of BM-MSCs, with Cytomix-treated cells performing best. (**B**) Similar to that of their BM counterparts, immunosuppressive function of AD-MSC was augmented by all preconditioning treatments; however, compared to other groups, the normoxia-treated cells were significantly more potent. (**C**) BM-MSC co-cultures resulted in increased levels of IL-1RA and IL-1β, while the secretion of the pro-inflammatory cytokines IL-6, IL-8, and IFN-γ was inhibited. (**D**) AD-MSC co-cultures resulted in increased levels of IL-1RA, while IL-4 and IFN-γ secretion was suppressed. * *p* < 0.05, ** *p* < 0.01, *** *p* < 0.001, **** *p* < 0.0001.

The immunosuppressive capacity of the different MSCs was evaluated using an MLR assay. Analysis of T-cell proliferation revealed that all groups were able to significantly suppress T-cell proliferation (*p* < 0.0001) compared to controls; however, preconditioning significantly enhanced the immunosuppressive effects of BM-MSCs compared to normoxia, with Cytomix treatment being the most effective (*p* < 0.0001) (Figure 6A).

Similar to BM-MSCs, AD-MSCs were able to significantly suppress T-cell proliferation regardless of preconditioning, as compared to controls (*p* < 0.0001). Similar to BM-MSCs, preconditioning with Cytomix further enhanced the immunosuppressive capacity of AD-MSCs when compared to non-Cytomix-treated (normoxia and hypoxia) groups (*p* < 0.0001). (Figure 6B).

To examine their immunomodulatory activity, cytokine secretion was also examined in the MLR co-culture system. In BM-MSCs, the secretion of IL-1RA was significantly increased under all conditions as compared to controls (*p* < 0.01). IFN-γ and IL-6 production were significantly decreased in all groups as compared to controls (*p* < 0.0001), with significantly enhanced IL-6 suppression following Cytomix-normoxia and hypoxia preconditioning, as compared to normoxia (*p* < 0.05). Conversely, secretion of IL-1β was significantly enhanced compared to controls (*p* < 0.01). IL-8 secretion was significantly suppressed (*p* < 0.001) following preconditioning as compared to controls. (Figure 6C).

Similar to that in BM-MSCs, secretion of IL-1RA was significantly increased in AD-MSCs co-cultures as compared to in controls. All groups significantly suppressed the secretion of IFN-γ as compared to controls (*p* < 0.0001). Similarly, IL-4 levels were significantly decreased under all conditions, but more significantly decreased (*p* < 0.001) following Cytomix treatment (Figure 6D).

## 4. Discussion

MSCs are attractive candidates for cell-based therapies. MSCs can be isolated from various sources. However, in more than half of the clinical trials to date, BM has been used as the source for MSCs, followed by umbilical-cord blood and adipose tissue [33]. At present, it is still unclear which source provides MSCs with superior functionality, but it has been shown that MSCs from different sources share some similar characteristics [2,34]. As the field advances, it is expected that the source of MSCs will likely be determined by the target indication and not by the availability or ease of isolation. For example, adipose-derived MSCs are reported to exhibit higher angiogenic and immunomodulatory capacity than BM-derived MSCs [5,35,36]. 

Aside from source, the therapeutic properties of MSCs can be affected by post-injury physiological factors, such as hypoxia and inflammation. It has been shown that exposing cells to these environments in vitro can augment their therapeutic capacity and potentially prepare or precondition them for in vivo use [30]. Przybyt et al. preconditioned AD-MSCs using a combination of hypoxia and IL-1β. They reported that CM from the preconditioned AD-MSCs enhance cardiomyocyte proliferation in vitro. To the author’s knowledge, the present study is the first to compare preconditioning methods in both BM-MSCs and AD-MSCs using a combination of hypoxia and Cytomix followed by a comprehensive assessment of MSC characteristics and therapeutic potency. Herein, we demonstrate that these preconditioning approaches are highly effective in augmenting the immunotherapeutic function of MSCs (Table 1).

To mimic an injurious inflammatory milieu, we utilized Cytomix, which contains IL-1β, IFN-γ, and TNF-α. Both IFN-γ and TNF-α have been shown to augment the immunomodulatory function of BM-MSCs and AD-MSCs [37,38]. To mimic hypoxia, we used a dedicated oxygen chamber and incubated the MSCs at 2% oxygen for 48 h, as we have previously found that these oxygen conditions are optimal for MSC function. 

In terms of cell phenotype, the preconditioned BM-MSCs and AD-MSCs retained high levels of positive MSC markers (above 97% and 93%, respectively); however, TF expression was elevated following exposure to Cytomix. Hypoxia treatment significantly increased the metabolic activity of BM-MSCs. This response to hypoxia has been well documented in the literature [39,40,41] and our results are in agreement with our previous study [30]. Furthermore, the increase in metabolic activity is in agreement with a recent report correlating increased metabolism with higher immunosuppressive capacity of BM-MSCs [42]. Morphological changes were observed after preconditioning cells with Cytomix. The MSCs became elongated, exhibiting a more spindle-like appearance. Notably, Klinker et al. recently reported that the morphology of MSCs can predict their overall immunosuppressive capacity [43]. In that study, they showed that thin elongated MSCs have a better immunosuppressive capacity than rounder MSCs, an observation that is consistent with our findings. Importantly, apoptosis has also been correlated with the immunosuppressive potency of MSCs. Galleu and colleagues reported that MSCs undergo apoptosis by recipient cytotoxic cells, a process that is essential for MSC-induced immunosuppression. They propose that apoptotic MSCs can be infused into patients to cause immunosuppression [44]. In this study, apoptosis was increased by the addition of Cytomix. In agreement with the aforementioned studies, the Cytomix-treated MSCs were also the most potent in effecting immunosuppression. Furthermore, the higher rate of apoptosis seen in the AD-MSCs correlated with higher immunosuppressive capacity, as compared to that of BM-MSCs (~20% vs. ~40% T-cell proliferation, respectively; Figure 6A,B). 

Evaluation of the differentiation capacity of the preconditioned MSCs revealed that the combination of hypoxia and Cytomix impairs their multipotent characteristics. Specifically, the BM-MSCs were unable to differentiate down the osteogenic pathway, while the AD-MSCs lacked chondrogenic and adipogenic potential. Similar findings were reported by both Wang et al. and Sullivan et al. In their studies, murine BM-MSCs treated with inflammatory mediators demonstrated reduced ability to differentiate down the osteogenic and adipogenic pathways [45,46]. 

MSC response to preconditioning was also measured via gene expression. *BCL-2*, *BAX*, *CYCS*, and *CASP3* genes encode proteins that are important factors in the apoptosis pathway. In this study, we observed an upregulation in *CASP3* expression in the Cytomix-hypoxia BM-MSCs, which is consistent with the high percentage of MSCs undergoing late apoptosis as shown by flow cytometry (Figure 2D). In AD-MSCs, we observed higher rates of apoptosis, particularly in the Cytomix-hypoxia group. Gene expression shows an inverse relationship between *BAX* and *BCL-2*, suggesting an anti-apoptotic signature. This may suggest that the cells are attempting to protect themselves from the harmful inflammatory (Cytomix-hypoxia) milieu. This observation is consistent with other studies demonstrating that *BAX*/*BCL-2* ratio is important in the regulation of apoptosis [47,48]. 

The *VEGF-A* protein is central to the formation of new networks of blood vessels [49]. Increased *VEGF-A* expression/secretion due to hypoxia (and hypoxia-inducible factor activation) is a known molecular pathway that has been extensively reported in the literature [40,41,50,51] and is also consistent with our previous findings [30]. In this study, AD-MSCs showed a much more significant upregulation of *VEGF-A* under hypoxia (Figure 3B). This could be due to inherent angiogenic disposition seeing that they were retrieved from the stromal vascular fraction. The results from the BM-MSCs reveal that *VEGF-A* protein secretion may increase following preconditioning, especially after exposure to both hypoxia and Cytomix (Figure 3A).

The *F3* gene encodes for TF, a cell surface glycoprotein that signals cells to initiate the blood coagulation cascade. Systemic administration of cells with increased TF expression may present a safety concern, such as pulmonary embolism. Since various groups have reported the expression of TF by MSCs under certain conditions [25,28,30,52], we wanted to assess its expression following preconditioning in both BM-MSCs and AD-MSCs. Flow cytometry data indicated no increase in TF expression due to hypoxia in human BM-MSCs, which is consistent with our previous work [30]. In contrast, TF expression increased dramatically when BM-MSCs were treated with Cytomix. Gene expression data support these findings with a significant upregulation in *F3* in the +2% BM-MSCs (*p* = 0.018). In contrast to in BM-MSCs, in AD-MSCs, TF surface expression measured via flow cytometry was increased under hypoxia and Cytomix treatment. These findings are further corroborated by significant upregulation of the TF gene under hypoxia and Cytomix as compared to that under normoxia (*p* < 0.0001). Finally, a direct correlation exists between the angiogenic genes *VEGF-A* and TF with a concomitant increase in both BM-MSCs and AD-MSCs [53,54].

Following preconditioning, we also observed an upregulation in *HMGB-1* and its receptor, *TLR-4*. This effect was more significant in AD-MSCs, which indicates that preconditioning promotes MSC activation, as TLR4 has been shown to play a key role in MSC response to injury [31], as well as MSC modulation of cytokine production [55]. Previous studies have shown that pro-inflammatory cytokines [56,57] and hypoxia [58,59,60,61] upregulate *HMOX1* expression, which was shown to play a key role in the immunosuppressive function of MSCs [62]. The anti-inflammatory function of *HMOX1* is also correlated with IL-1RA secretion [63]. In this study, *HMOX1* was upregulated in BM-MSCs following preconditioning with a concomitant increase in the secretion of IL-1RA, both in single and co-cultures (Figure 4A, Figure 5A, and Figure 6C). This relationship was not observed in AD-MSCs as *HMOX1* was downregulated following Cytomix treatment, irrespective of hypoxia. Since both *HMOX1* and *CAT* play pivotal roles in protecting cells from reactive oxygen species [64,65], their expression is correspondingly regulated and coupled following preconditioning in both BM-MSCs and AD-MSCs (Figure 3). *TNFAIP6* is implicated in mediating the anti-inflammatory and tissue protective functions of MSCs [66]. TSG-6 is a protein encoded by *TNFAIP6* that plays a vital role in MSC’s therapeutic capacity for resolving lung injury [67,68] and sterile inflammation [69]. In this study, *TNFAIP6* expression was significantly upregulated following Cytomix treatment in both BM-MSCs and AD-MSCs. Similarly, *STC-1* and *PTGS-2* were upregulated in both BM-MSCs and AD-MSCs following Cytomix treatment. The therapeutic effects of *STC-1* include reduction of oxidative stress, resolution of lung fibrosis, as well as attenuation of inflammatory response by monocyte and macrophage [70,71]. *PTGS-2* encodes for the cox-2 enzyme, which is responsible for the production of prostaglandin E2 (PGE2). PGE2 plays a key role in the immunosuppressive effects of MSCs [72,73]. Therefore, upregulation of *PTGS-2* following preconditioning is most likely correlated with the augmented MSC immunosuppression seen in this study (Figure 6A,B).

Since paracrine activity has been identified as a key function of MSCs, the secretion profile of MSCs was investigated in response to preconditioning. In single cultures, we observed robust secretion of IL-1RA, and to a lesser extent IL-10 following Cytomix treatment (Figure 4). Elevated levels of the anti-inflammatory cytokines IL-1RA and IL-10 are paramount for the resolution of ARDS [74,75].

Secretion profile was also assessed in an MSC–MNC co-culture following an LPS challenge in order to examine their inflammatory response as well as assessing donor variation (Figure 5). To perform this, we tested a total of 6 different donors (3 donors from each tissue). In both MSC types, significantly high levels of the anti-inflammatory cytokines, IL-1RA and IL-10, were evident following an inflammatory challenge with a more robust response following the Cytomix treatment. TNF-α has been implicated as an inflammatory mediator of ARDS/ALI [76,77]. Both types of MSCs were able to significantly inhibit the secretion of TNF-α but varied by donor. While the Cytomix treatment triggered higher levels of anti-inflammatory cytokines, it also significantly increased the secretion of the pro-inflammatory IFN-γ in both BM-MSCs and AD-MSCs, which is a key mediator for regulating the immunomodulatory function of MSCs [38,43]. In general, the MSCs responded to preconditioning similarly, regardless of the inherent donor variation.

An MLR assay was used to evaluate the immunomodulatory capabilities of the preconditioned BM-MSCs and AD-MSCs. In this assay, the MNCs are stimulated with a mitogen (PHA) to trigger T-cell proliferation. Both MSC types were able to significantly suppress T-cell proliferation; however, the Cytomix-treated groups were the most effective, with the +21% AD-MSCs eliciting the most potent response. Our results add to the existing body of literature illustrating the immunosuppressive effects of both BM-MSCs and AD-MSCs [78,79,80]. Evaluation of secreted factors demonstrated robust secretion of IL-1RA from both cell types, thereby validating once again that IL-1RA is central to the therapeutic action of MSCs.

Taken together, Cytomix treatment in combination with hypoxia culture resulted in MSCs with augmented therapeutic capacity, evident via enhanced functional characteristics as well as immunosuppressive and immunomodulatory functions. However, the combinatory treatment appeared too harsh/injurious for the cells, evident from the decrease in their multipotent potential, reduced clonogenic capacity, and increased *TF* expression. In contrast, Cytomix-treated MSCs cultured under normoxia showed a similar response in augmentation of function while concomitantly retaining their multipotent and self-renewal properties. It is important to note that the combinatory treatment may still prove useful under different conditions (e.g., different cytokine exposures, hypoxia tensions, and treatment windows). However, under these settings, we show that a single preconditioning approach, namely Cytomix treatment under normoxia, is ideal. 

The authors acknowledge that there are limitations in this study. Importantly, most assays were performed using a single donor per cell type and therefore these results could possibly arise from donor-dependent variations. However, in the anti-inflammatory assay, it was demonstrated that, using three different donors per cell type, the MSCs responded similarly to preconditioning, thereby suggesting minimal donor-dependent variation with preconditioning. This preconditioning method can be valuable for producing MSCs for anti-inflammatory indications, such as ARDS treatment. It is expected that these cells will exhibit superior function under harsh in vivo inflammatory conditions, which still needs to be proven. Clinical application of this approach can simply involve a short exposure time of AD-MSCs prior to cell infusion. However, it is important to note that in the case of AD-MSCs, thromboemboli could be of significant clinical consequence (as demonstrated by increased *TF* expression); therefore, for systemic cell administration BM-MSCs appear safer.

## 5. Conclusions

In this study, we evaluated various preconditioning methods, including the novel combination of hypoxia and Cytomix (TNF-α, INF-γ, and IL-1β) to determine their potential for augmenting the therapeutic function of MSCs in preparation for the harsh in vivo environment. Our data suggest that these preconditioning approaches can facilitate the production of MSCs with robust immunomodulatory and anti-inflammatory properties. In these settings, both BM-MSCs and AD-MSCs appear as promising candidates for the treatment of inflammatory conditions. BM-MSCs exhibit robust secretion capacity, while AD-MSCs elicit a potent immunosuppressive response. Taken together, Cytomix-treated AD-MSCs cultured under normoxia appear to be the ideal candidate for treating inflammatory conditions due to their retention of multi-differentiation potential and self-renewal capacity, robust anti-inflammatory properties, and potent immunosuppressive function. Nonetheless, safety concerns, such as thrombogenic disposition due to *TF* expression, should be carefully considered prior to clinical application.

## Figures and Tables

**Table 1:** Semi-quantitative summary of results. ↑ and ↓ denote increases and decreases, respectively. Additional arrows mean a larger magnitude of change (i.e., ↑ < ↑↑ < ↑↑↑ < ↑↑↑↑ and ↓ < ↓↓ < ↓↓↓ < ↓↓↓↓).

	21%		+21%		2%		+2%	
	BM	AD	BM	AD	BM	AD	BM	AD
**Gene Expression**								
***TF* (*F3*)**	-	-	-	↓↓	-	↑↑↑↑	↑↑	↑↑↑
***VEGF-A***	-	-	-	-	-	↑↑↑↑	-	↑↑↑↑
***BCL-2***	-	-	↑↑	↑	-	↑↑	-	↑↑↑↑
***HMOX1***	-	-	↑↑	↓↓	↑	-	-	↓
***TNFAIP6***	-	-	↑↑↑	↑↑↑	-	↑↑	↑	-
**MSC Secretion**								
**IL-1RA**	-	-	↑↑	↑	-	-	↑↑	↑↑
**IL-8**	-	-	↑↑↑↑	↑↑↑↑	-	-	↑↑↑↑	↑↑↑↑
**Immunosuppression (MLR Assay)**	↑	↑	↑↑	↑↑↑↑	↑	↑	↑↑	↑↑↑
**Immunomodulation (MLR Assay)**								
**IFN-γ**	↓↓↓↓	↓↓↓↓	↓↓↓↓	↓↓↓↓	↓↓↓↓	↓↓↓↓	↓↓↓↓	↓↓↓↓
**IL-1β**	↑↑	-	↑↑	-	↑	-	↑↑	-
**IL-6**	↓	-	↓↓	-	↓↓	-	↓	-
**IL-8**	↑↑↑↑	-	↓↓↓	-	↓↓↓	-	↓↓↓	-
**IL-1RA**	↑↑	↑↑	↑↑	↑↑	↑↑	↑↑	↑↑	↑↑
